# Matrix metalloproteinase-3 (MMP-3)–mediated gene therapy for glaucoma

**DOI:** 10.1126/sciadv.adf6537

**Published:** 2023-04-19

**Authors:** Jeffrey O’Callaghan, Conor Delaney, Merissa O’Connor, Joseph van Batenburg-Sherwood, Martin Schicht, Elke Lütjen-Drecoll, Natalie Hudson, Sorcha Ni Dhubhghaill, Peter Humphries, Chris Stanley, Annahita Keravala, Thomas Chalberg, Matthew S. Lawrence, Matthew Campbell

**Affiliations:** ^1^ Smurfit Institute of Genetics, Trinity College Dublin, Dublin 2, Ireland.; ^2^ Virscio Inc., New Haven, CT, USA.; ^3^ Department of Bioengineering, Imperial College London, London, UK.; ^4^ Institute of Functional and Clinical Anatomy, University of Erlangen-Nuremburg, Erlangen, Germany.; ^5^ Department of Ophthalmology, Antwerp University Hospital (UZA), Edegem, Belgium.; ^6^ Exhaura, 10 Earlsfort Terrace, Dublin 2, Ireland.

## Abstract

Approximately 80 million people globally are affected by glaucoma, with a projected increase to over 110 million by 2040. Substantial issues surrounding patient compliance remain with topical eye drops, and up to 10% of patients become treatment resistant, putting them at risk of permanent vision loss. The major risk factor for glaucoma is elevated intraocular pressure, which is regulated by the balance between the secretion of aqueous humor and the resistance to its flow across the conventional outflow pathway. Here, we show that adeno-associated virus 9 (AAV9)–mediated expression of matrix metalloproteinase-3 (MMP-3) can increase outflow in two murine models of glaucoma and in nonhuman primates. We show that long-term AAV9 transduction of the corneal endothelium in the nonhuman primate is safe and well tolerated. Last, MMP-3 increases outflow in donor human eyes. Collectively, our data suggest that glaucoma can be readily treated with gene therapy–based methods, paving the way for deployment in clinical trials.

## Teaser

Adeno-associated virus (AAV) vectors deliver MMP3 for the treatment of glaucoma.

## INTRODUCTION

Glaucoma in its various forms is the second most common cause of global visual loss after cataract. Current estimates suggest that up to 80 million people worldwide are affected by the disease (*1*). Up to 74% of glaucoma cases are of the primary open angle (POAG) form (*2*), with a 2.4% global prevalence of POAG in over 40 year olds (*3*), treatable using topically applied pressure-reducing medications. The cost of these medications per annum in the United States was recently estimated at approximately $1 billion (*4*). Between 25 and 50% of patients fail to achieve intraocular pressure (IOP) targets with first-line prostaglandin analogs, and up to 10% of patients (estimated at 6.4 million people globally) are suboptimally responsive to polypharmacy eye-drop treatments (*5*, *6*). Although multiple topical drops are efficacious for most patients when taken correctly, keeping up with polypharmacy dosing schedules is challenging, and compliance remains a serious and substantial problem that limits the real-world effectiveness of topical drops (*7*, *8*). While penetrating ocular surgery (minimally invasive glaucoma surgery/trabeculectomy/drainage tubes) is available for resistant cases, these surgeries are invasive and associated with complications. There is an urgent need for better clinical tools and approaches for these patients to prevent irreversible vision loss. Adeno-associated virus (AAV)–based gene therapy approaches have gained traction in ophthalmologic indications such as Leber congenital amaurosis, where Luxturna was approved by the U.S. Food and Drug Administration (FDA) in 2017 (*9*). In addition, gene therapies are also being deployed for other diseases where programs are advancing through development in both rare (choroideremia, achromatopsia, etc.) and common (age-related macular degeneration) diseases.

While the most common forms of glaucoma are not monogenic, we do know that IOP is maintained at physiological levels (an average of 16 mmHg with diurnal variation) in the normal eye as a result of a balance in the production of aqueous by the ciliary body and the resistance to its flow out of the eye, primarily through the conventional outflow pathway. This channel circumscribes the limbus, containing the trabecular meshwork (TM)—composed of a meshwork of extracellular matrix (ECM) surrounded by TM cells—and the inner wall of Schlemm’s canal (SC)—a wall of endothelial cells connected to the outer TM (juxtacanalicular), where the majority of outflow resistance is generated (*10*). A small proportion of aqueous humor also flows through the bundles of the ciliary muscles (supraciliary) or suprachoroidal spaces, comprising the so-called unconventional outflow pathway (or uveoscleral). In open-angle glaucoma, the SC inner wall and its basal lamina become more resistant to the flow of aqueous, resulting in a chronic elevation of IOP (*11*). This ultimately damages the optic nerve fibers and, if untreated, can lead to total irreversible blindness. While pressure-reducing eye drops are the first-line standard of care in glaucoma management, it is interesting to note that most of these topical medications either slow the rate of aqueous production or enhance its removal through the unconventional pathway. Here, we report the development of an AAV-delivered enzyme, i.e., matrix metalloproteinase-3 (MMP-3), that can efficiently increase outflow facility in the nonhuman primate (NHP) eye following intracameral injection.

MMPs are involved in a wide range of both physiological and pathological processes. The ability to modulate ECM material and other signaling factors is a central part of development and homeostasis, including the processes of embryogenesis, cell survival, angiogenesis, immune response, and wound healing. Conversely, disruptions to MMP regulation can result in developmental disorders, inflammatory or cardiovascular conditions, or cancers (*12*, *13*). This highlights the balance between MMPs and tissue inhibitors of metalloproteinase (TIMPs), where the balance can quickly be tipped in favor of an excess or insufficiency of ECM degradation. Many ocular diseases are believed to exhibit a pathophysiology related, in part, to this disruption in regular MMP activity (*14*). For example, significant reductions in MMP activity have been shown in the aqueous humor of glaucoma patients (*15*, *16*).

The use of MMPs in glaucoma treatment has been an area of interest for decades; however, only in recent years has it been proposed as a directed treatment using genetic vectors. The potential for aqueous outflow modulation by MMPs was first realized by perfusing human anterior segments with high doses of purified MMPs and observing increases in outflow facility (*17*). This finding coincided with the release of topical latanoprost for IOP lowering, a treatment that is in use today as a first-line therapy for glaucoma patients. Prostaglandin analogs including latanoprost are known mediators of MMPs, which is believed to be a key mechanism of IOP lowering in these treatments. Several studies have also shown increased MMPs in human cells or cynomolgus monkeys after prostaglandin treatment (*18*–*22*). Studies have also been conducted using inducible MMP-1 expression from an AAV in sheep, highlighting the attractiveness of this approach (*23*).

We have previously published a proof-of-concept study assessing the efficacy of an MMP-3–mediated gene therapy approach in wild-type (WT) mice (*15*). Here, we show that AAV9 efficiently transduces corneal endothelial cells in vitro and in vivo. In two distinct mouse models of glaucoma, we show that intracameral delivery of MMP-3 via AAV9 is efficacious at increasing outflow and decreasing IOP. We also show that persistent and long-term expression of MMP-3 is safe and well tolerated in the NHP eye and can increase outflow facility in vivo. Last, we show that MMP-3 increases outflow facility in donor human eyes, demonstrating the promise of this approach to treating open angle glaucoma in terms of clinical translation.

## RESULTS

### Codon optimization increases MMP-3 expression levels

**Fig. 1.**
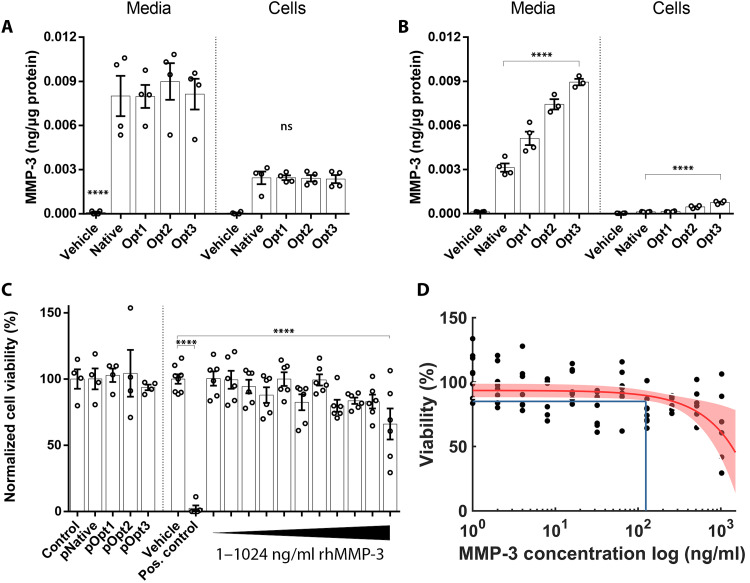
Codon optimization of expression vector. Codon optimization was applied to the native human MMP-3 sequence, and resulting plasmids were transfected into (**A**) HEK293 cells and (**B**) HCECs. An increase in MMP-3 protein expression was observed in HCECs from codon-optimized plasmids, particularly Opt3. (**C**) An MTS viability assay was performed on the codon-optimized plasmids and increasing doses of recombinant human MMP-3 (rhMMP-3) from 1 to 1024 ng/ml for 24 hours. A significant reduction in cell viability was detected at 1024 ng/ml. (**D**) An average viability of 85% was expected for HCEC with rhMMP-3 concentrations up to 130 ng/ml.

We have previously reported on the therapeutic potential of an AAV expressing MMP-3 to degrade ECM protein in the outflow pathway in mice (*15*). In this study, we aimed to maximize the expression of MMP-3 by optimizing the inverted terminal repeats (ITR)-flanking gene of interest. Initially, the plasmid backbone was improved by adding a Kozak sequence. Three plasmids with alternate codon usage were generated to test for increased transgene expression versus the WT sequence (table S1). These, along with the unoptimized control (native), were transfected into human embryonic kidney (HEK) 293 cells. Media and cell lysates were harvested 48 hours after transfection. No differences were observed between the optimized and native plasmid sequences in either the media or cell lysates of these cells [*P* = not significant (ns), *n* = 4; Fig. 1A]. However, upon transfection of these plasmids in the target cell, i.e., human corneal endothelial cells (HCECs), a clear increase in secreted protein expression was observed with the codon-optimized plasmids. The greatest elevation observed was in the Opt3 plasmid, giving a 2.85-fold increase over the native sequence (****P < 0.0001, *n* = 3 for Opt2 and Opt3, *n* = 4 for others; Fig. 1B). A similar pattern was also observed in cell lysates, with a sixfold increase observed with Opt3 over native (****P < 0.0001, *n* = 4; Fig. 1B). To further optimize expression, we altered various regulatory elements on the Opt3 cassette. These regulatory modifications are summarized in table S1. The resulting plasmids were transfected into HCECs. When analyzed by quantitative polymerase chain reaction (qPCR) (fig. S1A) and enzyme-linked immunosorbent assay (ELISA) (fig. S1B), respectively, no constructs demonstrated higher MMP-3 expression compared to the Opt3 construct; hence, these alternative regulatory elements were not used in future experiments. To determine the effect of these optimized constructs on corneal endothelial cell viability, an MTS assay was performed on transfected cells, with a plasmid carrying WT sequence used as a control to account for the effect of transfection. No significant difference was observed between any plasmids compared to the control (*P* = 0.82, *n* = 4; Fig. 1C, left). Increasing doses of recombinant human MMP-3 (rhMMP-3) were applied to HCECs for 24 hours from 1 to 1024 ng/ml to determine the point at which MMP-3 was toxic to cells. It was only at the highest dose of 1024 ng/ml that a significant reduction in cell viability was observed (***P < 0.001, *n* = 6; Fig. 1D, right). To define a conservative safety range for MMP-3 concentrations in HCEC media, these MTS data were interpolated to the lower confidence band of 85% viability, a value chosen for which we would expect anything lower to have a negative impact on the function of the cell monolayer. On the basis of the data in Fig. 1D, for concentrations below 130 ng/ml MMP-3, the average HCEC viability for *n* = 6 will exceed 85%.

### AAV-coMMP3 increases MMP-3 levels in vitro and in murine aqueous

**Fig. 2.**
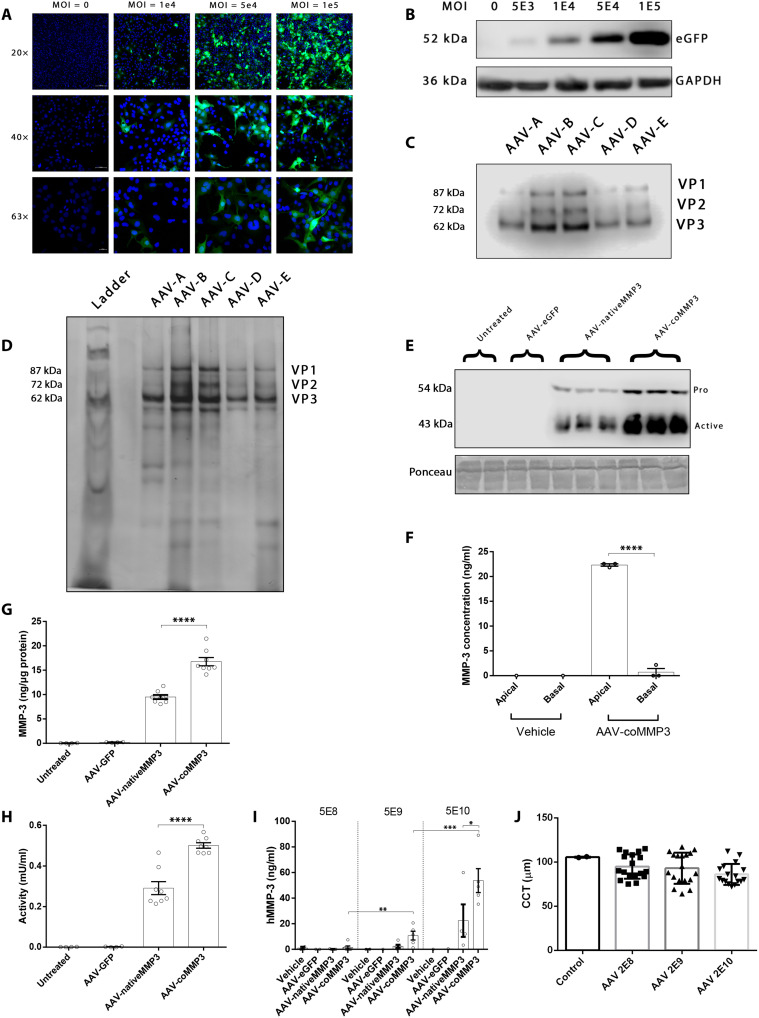
AAV development and validation. Quality control assays were performed on AAV stocks. (**A**) Immunocytochemistry was performed to visualize AAV transduction efficiency in HCECs. Scale bars, 100, 50, and 20 μm for 20×, 40×, and 63× images, respectively. (**B**) AAV expressing eGFP was added to HCECs at increasing MOIs and immunoblotted. Dose-dependent expression levels were observed. (**C**) Western blot for viral proteins. All AAVs express VP1, VP2, and VP3. (**D**) Silver stain of AAV stocks. The fourth lane represents the purest batch. (**E**) AAV expressing native or optimized MMP-3 was transduced into HCECs. Forty-eight hours after transduction, both pro and active forms of MMP-3 are elevated, especially in AAV-coMMP3. (**F**) HCECs were grown in Transwells and subjected to AAV-coMMP3 at an MOI of 1 × 10^5^. At 24 hours, nearly all the secreted protein was found expressed in the apical compartment. (**G**) MMP-3 protein levels and (**H**) activity were found to be significantly increased in the media of HCEC when treated with AAV-coMMP3 compared with AAV-nativeMMP3. (**I**) AAV expressing native or optimized MMP-3 was intracamerally injected into the anterior chamber of mice, and aqueous samples were collected 4 weeks later and assayed by ELISA. (**J**) Central corneal thickness was measured and found to not significantly differ between vector doses.

To examine the transduction efficiency of AAV9 in HCECs, AAV9 expressing enhanced green fluorescent protein (AAV9-eGFP) was added to monolayers at increasing multiplicity of infection (MOI) and GFP expression was determined after 48 hours (Fig. 2A). Images taken at 20× were quantified, with the percentage of cells expressing GFP thresholded at an arbitrary value significantly increasing at each dose [one-way analysis of variance (ANOVA) with Tukey’s multiple comparisons, ****P < 0.0001, *n* = 3]. Mean GFP fluorescent intensity was also significantly greater at 1 × 10^5^ MOI [75.9 normalized integrated gray level (IGL)] compared to 1 × 10^4^ (8.5 IGL) and vehicle (1 IGL, one-way ANOVA with Tukey’s multiple comparisons, **P = 0.004, *n* = 3). Cell lysates were then harvested and probed for GFP by Western blot (Fig. 2B). In both cases, a dose-dependent effect was observed, and transfection efficiency was high at greater MOIs. The Opt3 plasmid was then incorporated into an AAV9, while the native plasmid was also synthesized into AAV. First, the AAVs were titered by qPCR to verify the concentration. AAV-coMMP3 was determined to have a titer of 2.81 × 10^13^ vector genomes (vg)/ml, while AAV-nativeMMP3 had a titer of 2.62 × 10^13^ vg/ml. A Western blot was performed on several AAV-coMMP3 batches (lanes A to E), which demonstrated the expected banding pattern (Fig. 2C). A silver stain was also used to identify purity of the samples. The fourth AAV batch appeared to have the highest purity (Fig. 2D) and was used in subsequent experiments. To assess MMP-3 expression, HCECs were transduced, and media samples were taken after 48 hours, followed by Western blotting for MMP-3. Both the pro and active forms of MMP-3 were increased with AAV-coMMP3 compared to AAV-nativeMMP3 (Fig. 2E). To characterize MMP-3 secretion from HCEC, cells were seeded on Transwell plates under conditions where they grow as a polarized monolayer and treated with AAV-coMMP3. Media were sampled from both the apical and basal chambers at 24 hours. MMP-3 was secreted almost exclusively in the apical direction, indicating the polarization of these endothelial cells, and demonstrating that high levels of MMP-3 can be expressed from AAV9 (22.32 ng/ml, ****P < 0.0001, *n* = 3; Fig. 2F).

To verify codon optimization in AAV, HCEC monolayers were treated with AAVs and the media were harvested after 48 hours. MMP-3 levels were significantly increased in the media from HCEC treated with AAV-coMMP3 compared to those with AAV-nativeMMP3 (1.76-fold increase, ****P < 0.0001, *n* = 8; Fig. 2G). An activity assay was performed on the same media samples, and a similar expression pattern was observed (1.72-fold increase, ****P < 0.0001, *n* = 8; Fig. 2H). This suggests that the MMP-3 produced is being converted to its active form upon secretion.

Last, we sought to examine whether sequence optimization observed in primary HCEC cultures translated to higher expression in vivo. Mice were injected intracamerally with varying doses (5 × 10^8^, 5 × 10^9^, and 5 × 10^10^ vg) of AAVs, and aqueous was collected every 2 weeks. AAV-coMMP3 showed a strong dose response, with 5 × 10^9^ vg showing higher levels than 5 × 10^8^ vg (10.72 ng/ml versus 1.44 ng/ml, *P = 0.023, *n* = 5 and *n* = 6, respectively), and 5 × 10^10^ vg higher than 5 × 10^9^ vg (53.69 ng/ml versus 10.72 ng/ml, **P = 0.002, *n* = 5). Compared to AAV-nativeMMP3, AAV-coMMP3 expression trended higher at all three doses and reached significance at 5 × 10^10^ vg (53.69 ng/ml versus 22.42 ng/ml, *P < 0.05, *n* = 5 for AAV-coMMP3, *n* = 4 for AAV-nativeMMP3; Fig. 2I). The central corneal thickness did not change between control and increasing doses of AAV9 (*P* = ns, *n* = 2 for control, *n* = 18 for AAV treatments, Tukey’s multiple comparisons; Fig. 2J).

### AAV-mediated MMP-3 expression decreases IOP in two mouse models of glaucoma

**Fig. 3.**
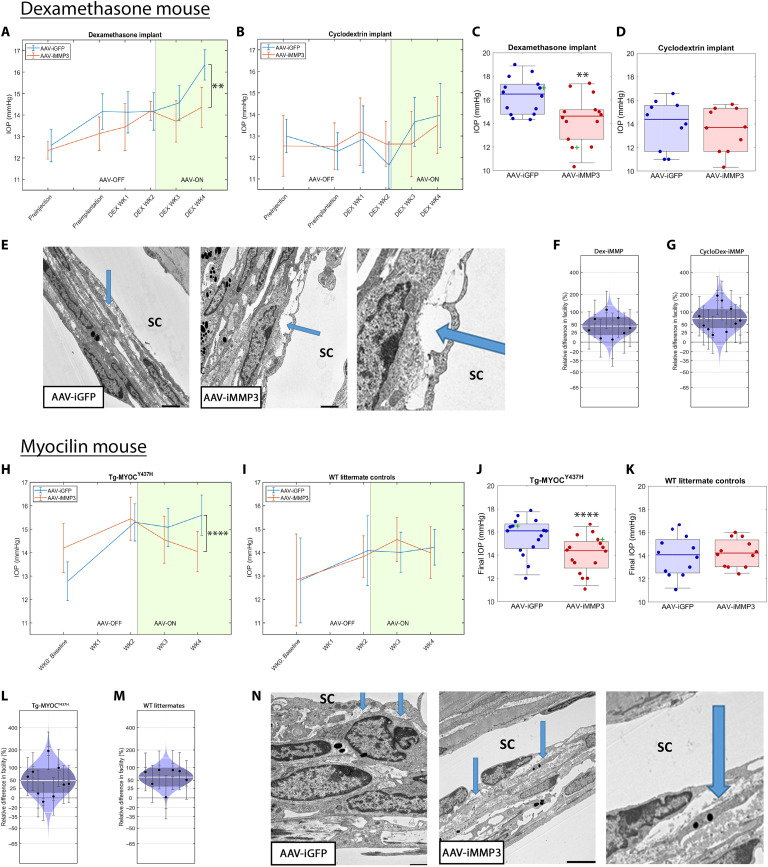
Proof of concept in mouse models of glaucoma AAV. iMMP3 was intracamerally injected into WT mouse eyes, with AAV-iGFP as a contralateral control. After 2 weeks, osmotic minipumps were subcutaneously implanted containing (**A**) dexamethasone or (**B**) cyclodextrin. IOP was measured for 4 weeks after implantation, and AAVs were induced using a doxycycline eye drop 2 weeks after implantation. Error bars indicate 95% confidence intervals; *n* = 14 and 10, respectively, for each time point. (**C** and **D**) The final time point was compared using boxplots, which showed a 1.9 median mmHg reduction of IOP in MMP-3–treated eyes only in the dexamethasone/hypertensive eyes. Whiskers represent the 5th and 95th percentiles. Green “+” sign indicates the eyes referenced in (E). (**E**) Transmission electron microscopy showed a dense JCT and subendothelial region rich with ECM (arrows), which was cleared in MMP-3–treated eyes (arrows). Scale bars, 2 μm. (**F**) Outflow facility was increased by 45% [18%, 78%] in treated dexamethasone-implanted mice. (**G**) Outflow facility was increased by 72% [38%, 115%] in cyclodextrin-implanted mice. AAVs were again injected as before into transgenic myocilin mice. (**H** and **I**) AAVs were induced 2 weeks after injection in both myocilin mice and WT littermates. Error bars indicate 95% confidence intervals; *n* = 16 and 12, respectively, for each time point. (**J** and **K**) At the final time point, AAV-iMMP3 eyes had an IOP 1.7 mmHg lower than controls, again only in hypertensive animals. Green “+” sign indicates the eyes referenced in (N). (**L**) Outflow facility was increased by 49% in treated myocilin mice (**M**) and by 61% in normotensive controls (**N**). According to electron microscopy images, greater ECM at the subendothelial region was found in myocilin eyes (arrows) compared to littermate controls, and flow channels devoid of ECM (arrows) were found regionally in MMP-3–treated eyes. Left scale bar, 2 μm; right scale bar, 5 μm.

For the purposes of the murine studies, an AAV vector consisting of native murine MMP-3 was created with a tetracycline-inducible promoter. A control AAV expressing inducible GFP was used for contralateral control eyes. Two mouse models were used: a dexamethasone-induced ocular hypertension and transgenic myocilin mouse. Animals were injected intracamerally using previously established protocols (*24*). In the first model, osmotic minipumps were filled with either dexamethasone or cyclodextrin and subcutaneously implanted 2 weeks after AAV injection. Minipumps were left in place for an additional 4 weeks, the extent of the pump’s capacity. Two weeks after implantation, topical doxycycline eye drops were applied twice daily to induce transgene expression. Cyclodextrin vehicle control animals did not exhibit significant increases in IOP (median change of 1.5 mmHg, *P* = 0.25, *n* = 10; Fig. 3B). In dexamethasone-treated animals, IOP gradually increased over the course of the exposure to dexamethasone, with a median IOP increase of 3.5 mmHg in control eyes (AAV-iGFP) compared to the baseline time point (****P = 0.0001, *n* = 14; Fig. 3A). AAV-iMMP3–treated eyes increased similarly to AAV-iGFP–treated controls for the first 2 weeks before transgene induction. After doxycycline eye drops were initiated, IOP in AAV-iMMP3–treated eyes decreased and, by the final time point at 6 weeks after injection, was 1.9 mmHg lower than in contralateral AAV-iGFP–injected controls (**P = 0.002, *n* = 14, Wilcoxon signed-rank test; Fig. 3C). Conversely, IOP was not significantly lowered in normotensive, cyclodextrin control animals (0.7 mmHg, *P* = 0.71, *n* = 10; Fig. 3D). Additional statistical tests on IOP measurements are described in Materials and Methods.

Transmission electron microscopy showed juxtacanalicular tissue (JCT) and subendothelial regions dense with ECM in control eyes, whereas there was an increase in optically empty spaces indicating ECM degradation in AAV-iMMP3 eyes (Fig. 3E). These optically empty spaces were quantified in fig. S2A. A zoomed-in image is provided to the right of Fig. 3E to highlight the empty spaces of the subendothelial region posttreatment. Outflow facility was increased on average in both hypertensive and normotensive eyes in response to AAV-iMMP3 compared to those treated with AAV-iGFP {45% [18%, 78%] (mean [95% confidence interval]), ***P = 0.0049, *n* = 8, Fig. 3F, and 72% [38%, 115%], ****P = 0.0004, *n* = 10, Fig. 3G, respectively}.

In the second model, we used the Y437H myocilin mouse, which has been previously described (*25*). Four-month-old Tg-MYOC^Y437H^ animals exhibited a higher IOP than that of WT littermate controls (1.9 mmHg, *P* = 0.01, *n* = 16 for Tg-MYOC^Y437H^, *n* = 12 for WT; Fig. 3, H and I, blue lines). At the final time point 4 weeks after injection, IOP was significantly reduced in the AAV-iMMP3–treated eyes of hypertensive animals by 1.7 mmHg (****P = 0.0003, *n* = 16, Wilcoxon signed-rank test; Fig. 3J); however, no reduction was observed in normotensive mice (*P* = 0.48, *n* = 12; Fig. 3K). Additional statistical tests on IOP measurements are described in Materials and Methods. Outflow facility was increased in response to AAV-iMMP3 by 49% [13%, 97%] and 61% [30%, 100%] on average in hypertensive (*P = 0.012, *n* = 9; Fig. 3L) and normotensive animals (**P = 0.002, *n* = 7; Fig. 3M), respectively.

To understand morphological changes in the Tg-MYOC^Y437H^ model, we first examined the ultrastructure of the outflow pathway in control eyes for contextual reference. The length of the SC was found to vary about the circumference to a much greater extent in Tg-MYOC^Y437H^ than WT mice, and we quantified the filtering area as a percentage of the SC length, which was significantly reduced in Tg-MYOC^Y437H^ mice (*P = 0.02, *n* = 4; fig. S2B). The presence of subendothelial ECM was also quantified as a percentage of the inner wall length and was significantly elevated in these mice (*P = 0.02, *n* = 4; fig. S2C). Activated cells, or those producing ECM, proliferating, or containing a large number of rough endoplasmic reticulum (rER) and Golgi, were quantified throughout the outflow tissue. An example of an activated cell is portrayed in fig. S2H. There was a greater number of activated cells as a proportion of total cells in Tg-MYOC^Y437H^ outflow tissue (**P = 0.01, *n* = 4; fig. S2D). Figure S2 (E to I) shows representative images of these mice indicating the presence of several interesting features including (fig. S2E) an accumulation of ECM material in the subendothelial region, (fig. S2F) thickening of the elastic fiber sheath, (fig. S2G) a dense band-like TM and JCT containing collagen deposits and nearly no optically empty spaces, (fig. S2H) activated cells with large numbers of enlarged rER cisterna and Golgi apparatus, and (fig. S2I) an increase in subendothelial cells.

Figure S2 (J to L) depicts semi-thin sections of WT, Tg-MYOC^Y437H^ control, and Tg-MYOC^Y437H^ treated with AAV-iMMP3, respectively. Abnormally small SC with an ECM-dense TM is noted in many Tg-MYOC^Y437H^ sections, while in treated eyes there are areas with loosened TM and, in some cases, peripheral retinal cysts presumably indicating an increase in uveoscleral outflow. Tracer studies were used to visualize the outflow pathway of WT (fig. S2M) and Tg-MYOC^Y437H^ (fig. S2N) eyes, and there appeared to be a decrease in high flow regions and an increase in low flow regions on average across groups; however, this was not statistically significant (~3 regions difference, *P* = 0.18, *n* = 4; fig. S2O). Of the MMP-treated Tg-MYOC^Y437H^ eyes examined, approximately half exhibited a loosely arranged TM with increased optically empty spaces in the JCT (Fig. 3N), whereas the other half showed widened spaces in the ciliary muscle–uvea region and increased peripheral retinal cysts, indicating that either the conventional or unconventional outflow pathway can be enhanced to reduce IOP in this model. A zoomed-in image is provided to the right of Fig. 3N to highlight the empty spaces of the subendothelial region posttreatment.

### Recombinant MMP-3 increases outflow in nonhuman primates in vivo

**Fig. 4.**
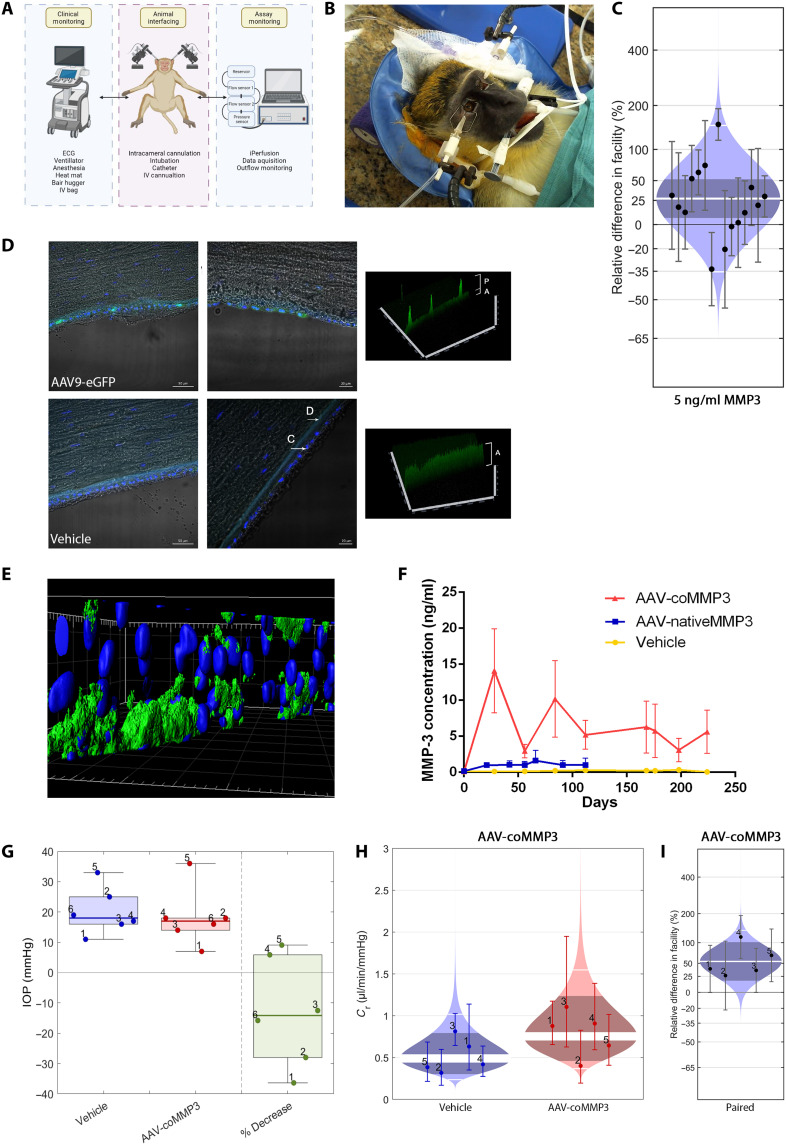
In vivo outflow facility measurements and AAV9 transduction in nonhuman primates. (**A**) Diagrammatic representation of the experimental setup. Anesthetized animals were carefully monitored and stabilized, while iPerfusion was used to record outflow measurements. IV, intravenous. (**B**) Representative image of a cannulated primate during the procedure. (**C**) Outflow facility was statistically increased by 27% [6%, 52%] on average following intracameral infusion of rhMMP-3 (5 ng/ml) in one eye for 1 hour. (**D**) NHPs were intracamerally injected with AAV9 expressing eGFP. After 64 days, eyes were enucleated and assayed for GFP expression by immunohistochemistry. Left scale bars, 50 μm; right scale bars, 20 μm. C, corneal endothelium; D, Descemet’s membrane; A, autofluorescence; P, positive signal. (**E**) 3D render of a z-stack through the corneal endothelium. Blue = nuclei, green = GFP. GFP was found to be localized exclusively to the corneal endothelium. (**F**) MMP-3 concentration determined by ELISA in the aqueous humor of AAV-injected NHP eyes over time. AAV-coMMP3 provides significantly increased protein concentration compared to AAV-nativeMMP3. (**G**) Pre-perfusion tonometric IOP at 7 months. IOP was reduced on average by 13%. (**H**) Treatment group comparison of outflow facility values. (**I**) Paired percentage differences in outflow facility between eyes of each animal. There is an average 54% [18%, 102%] increase in facility in response to AAV-coMMP3 at 7 to 10 months.

Although murine models are useful for initial preclinical proof of concept, for clinical translation, the NHP eye offers a far superior model, sharing almost identical anatomical features to the human eye and a more similar immune system to assess potential safety of gene therapy vectors. Therefore, a study was initiated in NHPs to initially identify whether there was a modulating effect of MMP-3 on outflow facility. The experimental setup is shown in Fig. 4 (A and B). After anesthesia, monitoring systems were set up, and both eyes of the animal were intracamerally cannulated to the iPerfusion system. This system has been used routinely for murine eyes ex vivo and was adapted for use in larger in vivo eyes. An initial measurement of IOP was recorded, and then rhMMP-3 (5 ng/ml) was perfused into the anterior chamber for 1 hour. To facilitate delivery, perfusions were undertaken at a pressure of 5 mmHg above the resting IOP. Contralateral eyes were perfused simultaneously with vehicle control. An outflow facility measurement was then taken, after which a sample of the aqueous humor was extracted and retained for MMP-3 quantification. Facility measurements were taken from 15 animals, and paired differences were plotted on a cello plot. On average, a 27% [6%, 52%] increase in outflow facility was observed after a 1-hour treatment (**P = 0.01, *n* = 15; Fig. 4C).

### AAV9 demonstrates tropism for corneal endothelial cells in the NHP anterior segment

To assess whether AAV9 could efficiently transduce corneal endothelial cells in NHPs, AAV9-encoding eGFP was intracamerally injected into NHPs and examined 100 days after injection. Eyes were then enucleated and cryosectioned to prepare for immunohistochemistry. In eyes receiving AAV9-eGFP (5 × 10^11^ vg), the GFP signal was detected exclusively at the corneal endothelium, with no expression observed in other anterior segment tissues, including lens, iris, TM, SC, or ciliary body (see fig. S3 for representative images of these regions). Strong punctate signal was observed throughout this region (Fig. 4D, top left); however, when viewed with higher magnification, signal was present to some degree in most corneal endothelial cells in the section (Fig. 4D, top right). This signal was clearly differentiated from the autofluorescence of the Descemet’s membrane. No signal was observed in vehicle-treated eyes. Three-dimensional (3D) rendering of z-stacks demonstrated the extent of GFP expression in the corneal endothelium (Fig. 4E). A subset of animals was subsequently injected with AAV9 expressing MMP-3. Ocular health was monitored throughout the study (fig. S4). Corneal health measures included pachymetry and specular microscopy, techniques that are used clinically to determine central corneal thickness and corneal endothelial cell health, respectively. Two-way ANOVA analyses found no significant difference either between AAV and vehicle treatments or across a single treatment for the duration of the study in both cases (*P* = 0.44 and *P* = 0.55, respectively, *n* = 1 for vehicle, *n* = 3 for AAV; fig. S4, A and B). For inflammatory cell area and density measurements, no significant difference was found between AAV and vehicle treatment over the course of the study (cell area *P* = 0.81, cell density *P* = 0.79, *n* = 1 for vehicle, *n* = 3 for AAV; fig. S4, C and D). No damage was observed to the anterior chamber or cornea specifically, and iridocorneal angles remained open (fig. S4, E to G). Clinical inflammatory scores were low for the entire study (fig. S4H).

### AAV-mediated MMP-3 expression increases outflow in NHP

In a second study, six NHPs were intracamerally injected with AAV-coMMP3 or vehicle and detailed safety measurements were performed for 12 months. A total of 21 tolerability measures were performed throughout the year and scored according to severity using the Hackett-McDonald scoring method (*26*). Overall, AAV-coMMP3 was well tolerated in all eyes (fig. S5). Specular microscopy and pachymetry measures were also used to assess the integrity of the corneal endothelium. In all cases, endothelial integrity did not significantly change throughout the study (fig. S5, B to G). Aqueous humor was collected at regular intervals in eyes injected with AAV-nativeMMP3, AAV-coMMP3, or vehicle and was used to determine MMP-3 concentration by ELISA. The grand mean AAV-coMMP3–mediated MMP-3 expression was significantly greater than the vehicle signal across the study (mean expression in vehicle-injected eyes = 0.22 ng/ml, mean expression in AAV-coMMP3–injected eyes = 5.9 ng/ml, ***P = 0.003; Fig. 4F). IOP readings were taken immediately before perfusion from animals injected with AAV-coMMP3. In these normotensive animals, a trend toward IOP reduction of 2 mmHg was observed (13% decrease; *P* = 0.2, *n* = 6; Fig. 4G) but was not statistically significant, similar to our findings in normotensive mice. In vivo outflow facility measurements were taken using iPerfusion at 7 to 10 months after injection. Control and treated eyes were grouped, with control eyes having an average facility of 0.49 μl/min per mmHg, and treated eyes having an average facility of 0.72 μl/min per mmHg (*n* = 5 for both groups; Fig. 4H). One animal was excluded because of unstable flow measurements, caused by improper cannulation. Pairwise analysis was performed to determine the percentage difference in the facility between the eyes of each animal. As denoted in the cello plot, outflow facility was increased by 54% [18%, 102%] on average after AAV-coMMP3 treatment (**P = 0.01, *n* = 5; Fig. 4I).

### Recombinant MMP-3 increases outflow in human donor eyes

**Fig. 5.**
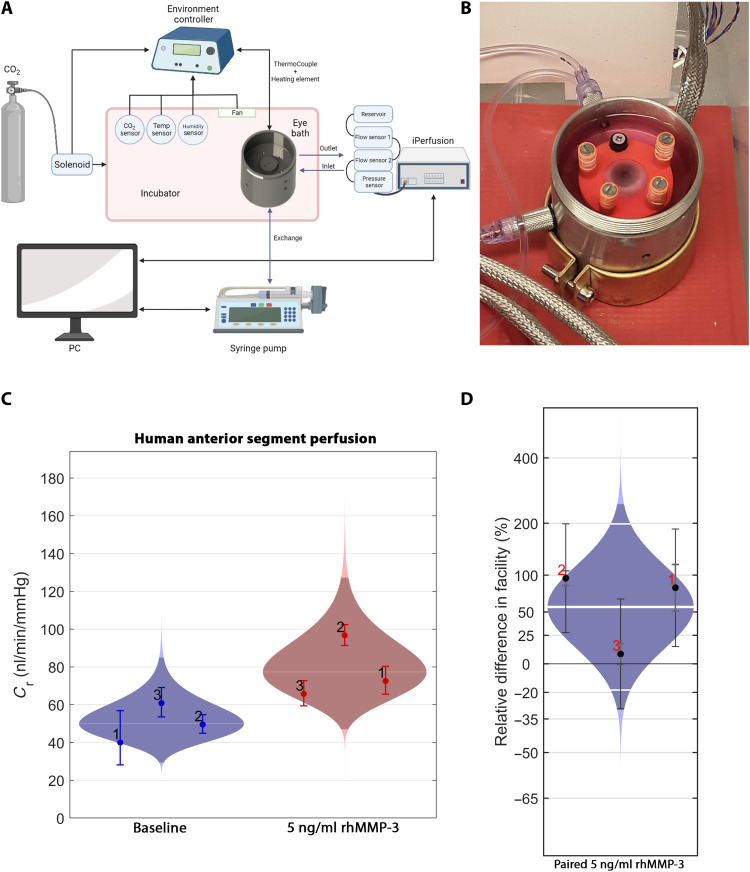
rhMMP-3 increases outflow in human donor eyes. (**A**) Diagrammatic representation of the iPerfusion system modifications to allow for the repeated measurement of outflow facility in human anterior segments. (**B**) Representative image of a human donor anterior segment mounted to the bespoke eye bath within the purpose-built incubator of the iPerfusion system. (**C**) Anterior segments were connected to the system and were allowed to reach a stable flow rate before a measurement was taken (blue). The media were exchanged with fresh media containing rhMMP-3 (5 ng/ml), and another measurement was taken after 1 hour (red). (**D**) Paired percentage differences of before and after MMP-3 treatment from the same eye. On average, there is a 56% difference in facility with these conditions.

To assess translatability of MMP-3–mediated increases in outflow facility to human eyes, further modifications were made to the iPerfusion system to allow perfusion of human donor anterior segments. An incubator was made for this purpose to cater for both environmental control and the numerous fluidic lines required. A simplified diagram of the setup is presented in Fig. 5A. An example of a human anterior chamber clamped to the chuck of the eye bath (excluding the cap), with heating element and fluid lines connected, is shown in Fig. 5B. Anterior segments were connected to the system, submerged in media, and allowed to stabilize at 12 mmHg before an outflow facility measurement was taken. The anterior chamber was then exchanged with either rhMMP-3 (5 ng/ml) or vehicle at a rate of 4 ml/min, for a total of 6 ml, to account for the volume of the anterior chamber and intermediate fluid lines. An outflow measurement was again taken and compared to the preceding baseline reading. On average, outflow facility increased by 56% after infusion with MMP-3 (*n* = 3 eyes; Fig. 5, C and D).

## DISCUSSION

Uncontrolled pressure in glaucoma remains a major clinical problem, with a significant number of patients who experience irreversible vision loss (*6*, *27*). This study represents the first gene therapy–based approach to IOP lowering that has been applied to primates based on a detailed knowledge of the underlying cause of the disease. First-line care for open-angle glaucoma patients involves the use of self-administered topical IOP-lowering formulations, the most widely used at present including prostaglandin analogs, carbonic anhydrase inhibitors, and β-blockers. As mentioned previously, none of these primarily target the major outflow channel, the TM. Therefore, it is highly likely that drugs directly targeting the TM would be developed and, the first of these, Rhopressa has recently been U.S. FDA-approved (*28*). Our strategy, which directly targets the outflow tissue, is unique in that problems with patient compliance (an important issue with all topical medications) are avoided and chronic self-administration is not required. Intracameral injection of AAV is amenable to an outpatient procedure in clinic, and repeated dosing may not be necessary given that AAV is known to express long term in nondividing tissues (*29*). In addition, intracameral injections are routine and safe and do not require the complex operating room infrastructure required to perform subretinal injections.

Following intracameral injection of a range of AAV serotypes, we were intrigued to observe that AAV9 had an exclusive tropism for the corneal endothelium. The putative mechanism for AAV9’s ability to bypass the blood-brain barrier is through the interaction of apically expressed glycoproteins on the endothelial cell surface. This is likely the mechanism by which it robustly transduces the corneal endothelium. Note that corneal endothelial cells are highly polarized, releasing MMP-3 exclusively on the apical aspect of the cell. The enzyme then flows with the natural current of aqueous into the TM and outflow channels, where it digests some of the ECM, rendering the tissue more permeable to aqueous flow. This also speaks to our data showing an early large increase in MMP-3 expression mediated by the AAV, which subsequently drops as the aqueous outflow increases. This will be a critical feature of the gene therapy in patients with advanced open angle glaucoma, where it is likely that the TM has become fibrotic with the deposition of excess ECM material.

We were able to detect elevated levels of MMP-3 in the anterior segment up to 200 days after injection, suggesting that a single injection will provide persistent and high levels of protein. Our observation that AAV does not induce ocular inflammation when injected intracamerally in NHP suggests promise for clinical translation. This finding stands in contrast to intravitreal AAV, which is associated with dose-dependent inflammation in both NHPs (*30*, *31*) and humans (Adverum program, National Eye Institute X-linked retinoschesis program). The relative immune privilege of the anterior chamber (anterior chamber-associated immune deviation: ACAID) may be more similar to the subretinal space; in both spaces, fluid is cleared from the eye relatively quickly, compared to relatively low turnover and therefore long residence times of vector in the vitreous. Last, the ability to detect increased outflow facility with MMP-3 in donor human eyes is a critical step in translating our findings to the clinic.

While our studies were conducted in the normotensive NHP eye and donor human tissues, it remains to be seen whether the approach will increase outflow and decrease IOP in the glaucomatous primate or human eye. However, we have observed decreases in IOP using this method in well-established murine models of glaucoma, suggesting that the biological mechanism is conserved across species. IOP measurements by rebound tonometry are inherently variable, and to address this limitation, we have taken readings over the course of 8 min and interpolated all data to 5 min after the onset of isoflurane anesthesia to account for the IOP decrease typically seen after anesthesia. We have also ensured that measurements were taken at the same time of day, ambient temperature, angle and position of probe, and anesthetic dose and performed by the same investigator. These studies highlight that MMP-3–mediated IOP changes are readily detected in hypertensive but not normotensive animals, although outflow facility changes are observed in both. We have additionally presented our findings on the morphology of the myocilin mouse model, which indicates underperfusion of the TM, increased ECM, regional shortening of the SC, and cellular proliferation. While MMP-3 digests ECM and increases conventional outflow in the dexamethasone model, this is the case with half of the myocilin model eyes tested, increasing unconventional/uveoscleral outflow in the remainder. We hypothesize that due to the altered TM, genetic defects leading to SC occlusion combined with elevated ECM and blocked channels, MMP-3 is likely being directed along the ciliary muscle. Where MMP-3 cannot physically act upon the conventional outflow tissue, it may instead regulate outflow through changes to the ECM in the uveoscleral route; this may occur in eyes with compromised TM structures, such as in older fibrotic eyes..

Glaucoma is the ideal disease candidate for genetic intervention in that it will remove patient compliance issues, the anterior chamber being readily accessible for local delivery, and AAV-mediated gene expression persisting long term and being well tolerated. Overall, our gene therapy–based approach to treating open angle glaucoma represents a method aimed at increasing the conventional outflow pathway in the eye to reduce IOP. Advancing therapeutic interventions for this devastating form of blindness, while progressing the era of gene therapies for common diseases, has the potential to have a profound impact on this important clinical need.

## MATERIALS AND METHODS

### Cell culture

HCECs (HCEC-B4G12) were purchased from Deutsche Sammlung von Mikroorganismen und Zellkulturen (DSMZ) (ACC 647) and cultured in human endothelial serum-free media (Invitrogen) with fibroblast growth factor 2 (FGF-2; 10 ng/ml) in a 5% CO_2_ incubator at 37°C. Cells were passaged with trypsin-EDTA (Gibco-BRL) and seeded into 24-well plates or 6-well Transwells (Corning) at a seeding density of 1.6 × 10^5^ cells/ml for treatment. Cells were used between passages 2 and 4 to ensure that cellular characteristics were retained.

HEK293 cells were purchased from the American Type Culture Collection (ATCC) (CRL-1573) and cultured in Eagle’s minimum essential medium (ATCC, 30-2003) with 10% fetal bovine serum.

Plasmids were transfected at 70 to 90% confluency. On the day of transfection, media were refreshed and plasmids were transfected using Lipofectamine 3000 (Invitrogen) with 500 ng of DNA and 1.5 μl of Lipofectamine per well. AAVs were added to cells at 70 to 80% confluency in 500 ml of media in 24-well plates at an MOI of 1 × 10^5^ unless otherwise stated.

### Cell viability

Cultured cells were treated with twofold increasing concentrations of rhMMP-3 (Abcam, ab96555) from 1 to 1024 ng/ml. Cell viability was assessed 24 hours after treatment using a CellTiter 96 AQueous One Solution Cell Proliferation Assay (Promega). Cells were incubated at 37°C for 1 hour with the supplied reagent, which was then transferred to a 96-well plate for reading by spectrophotometry (Multiskan FC, Thermo Fisher Scientific) at 450 nm. As presented previously, a modified approach was taken to determine at which concentration HCECs show a reduced tolerability to MMP-3. This was defined at an average of 85% viability over six cell samples. This conservative value ensures that a cell population would remain viable and still be able to proliferate. Anything lower should be regarded as MMP-3 intolerability, i.e., reduced cell proliferation or cell death. Control samples [phosphate-buffered saline (PBS) vehicle] were normalized to 100% viability, and a linear model was fitted to the normalized data and presented on a log *x* axis. The MMP-3 concentration at which cells had an average of 85% viability was interpolated from the lower 95% confidence bound from this linear model. This value represents the concentration of MMP-3 at which the average of six cell samples would have a 97.5% chance of retaining a greater to or equal than 85% viability.

### Plasmids and AAVs

Plasmids were designed and sequences were sent to ATUM DNA2.0 for synthesis. AAV plasmids containing AAV2 ITRs, a cytomegalovirus promoter, Kozak sequence, poly(A) tail, kanamycin resistance, and the human MMP-3 sequence were generated. Codon optimization was determined using proprietary algorithms. After plasmid selection by in vitro screening, plasmids were sent to Signagen Laboratories for incorporation into AAV9. AAV9 expressing eGFP was purchased from Signagen directly. Upon receipt of AAVs, aliquots were made and stored at −80°C. The following table summarizes the AAV vectors and their expression sequences used in this study (Table 1). Further details on plasmids used and their composition are described in table S1.

**Table 1.** AAV vector details.

AAV	Description
AAV9-nativeMMP3	Unoptimized human MMP-3 sequence
AAV9-coMMP3	Optimized human MMP-3 sequence
AAV9-eGFP	AAV expressing GFP
AAV9-iMMP3	Inducible murine MMP-3 sequence
AAV9-iGFP	Inducible GFP sequence

### AAV quality control assays

Standardized procedures were set up to ensure consistency across different AAV lots and used for all new AAVs before experimentation. To calculate titer for each AAV vector, a qPCR method was used with the use of a standard curve that was generated using linearized plasmid DNA, the same that was used to package into the vector. Primer probes to a specific sequence in this linearized plasmid were used for the qPCR assay. Titers were calculated by first digesting AAV with deoxyribonuclease and then proteinase K, which was heat-inactivated after 1 hour at 52°C. Reactions (20 μl) were performed in quadruplicate using FAM-MGB TaqMan gene expression assay kits designed to recognize the transgene and TaqMan universal master mix. Thermal cycling was performed on a StepOne Plus instrument with an initial polymerase activation of 10 min at 95°C, and then 40 cycles of 15 s at 95°C and 1 min at 60°C. Titer was determined by interpolating to the standard curve and multiplying by the dilution factor. Titers generally fell within twofold of the manufacturer-determined titer.

To determine vector purity and integrity, a silver stain was performed using the SilverQuest kit (Invitrogen) according to the manufacturer’s protocols. AAV (2 × 10^10^ vg) was boiled at 95°C for 10 min and loaded per lane of a 10% SDS–polyacrylamide gel electrophoresis (PAGE) gel. All buffers were made with ultrapure water, and care was taken to ensure that all equipment was washed in ultrapure water to reduce background noise. After staining, gels were transferred to an acetate sheet for scanning.

Western blot for viral proteins was performed in tandem with silver stain. Again, 2 × 10^10^ vg of AAV was loaded on a 10% SDS-PAGE gel and run at 90 V for 90 min. Membranes were blocked for 1 hour at room temperature in 5% nonfat dry milk and incubated overnight at 4°C with a mouse primary antibody to viral proteins (VP1, VP2, and VP3). Blots were washed 3 × 5 min in tris-buffered saline (TBS) and incubated at room temperature for 2 hours with horseradish peroxidase–conjugated anti-mouse secondary antibody (Abcam). Blots were developed on a blot scanner (C-Digit, LI-COR).

### Animals

Mice used in this study were carried out in accordance with regulations set out by The Health Products Regulatory Authority (HPRA), responsible for the correct implementation of EU directive 2010/63/EU. Ten-to-12-week-old male and female C57BL/6 mice were used in all experimentation outlined in this study as previously described (*32*). Mice were bred and housed in specific pathogen–free environments in University of Dublin, Trinity College, and all procedures complied with the HPRA project authorization number AE19136/P115.

### Intracameral injection

Intracameral injection was performed in mice as previously described (*24*, *32*). Animals were anesthetized by exposure to 3% isoflurane in oxygen. Pupils were dilated, and 2 μl of AAV vector was backfilled into a glass needle connected to a Hamilton syringe. An additional 1 μl of air was then withdrawn, and animals were injected intracamerally 1 to 2 mm anterior to the limbus with an empty needle to remove aqueous. Needles containing virus were guided toward the injection site using a micromanipulator, and the virus and air bubble were infused. The needle was left in the eye for 2 min to allow for equilibration before it was slowly withdrawn. Fucithalmic (Amdipharm) antibacterial was placed on the anterior surface, and animals were allowed to recover.

For injection into NHPs, an eye speculum was placed in the eye to facilitate injection followed by a drop of 0.5% proparacaine hydrochloride, then 5% Betadine solution, and a sterile saline rinse. Intracameral injections were performed with AAV vector or vehicle control in the contralateral eye. Injections were performed using a 0.3-ml insulin syringe with a 31-gauge needle. The needle was introduced through the temporal cornea, approximately 2 mm anterior to the limbus without disturbing intraocular structures. Following both intracameral injections, topical triple-antibiotic neomycin, polymyxin, bacitracin ophthalmic ointment was administered.

### Micro-osmotic pump implantation

Dexamethasone micro-osmotic pumps (model 1004, Alzet) were implanted as described previously (*33*, *34*). The dexamethasone was water soluble (D2915; Sigma-Aldrich), contained cyclodextrin (1.36 g per 100 mg of dexamethasone), and was dissolved in PBS. Vehicle-treated mice received pumps containing cyclodextrin alone. Dexamethasone was delivered at 2 mg/kg per day. Adult C57BL/6J mice of 10 to 12 weeks of age were anesthetized with 3% isoflurane at 1 liter/min, and surgical site was shaved and disinfected with chlorhexidine swabs. Mice were injected intramuscularly with buprenorphine (0.05 mg/kg) (Buprecare, Animalcare) and subcutaneously with enfloroxacine (5 mg/kg) (Enrocare, Animalcare). An incision was made between the scapulae, a subcutaneous pocket was created by blunt dissection, and the pump was inserted. The incision was closed using surgical glue (Surgibond, RayVet). After pump implantation, mice were housed individually, and diet was supplemented with Complan to prevent glucocorticoid-induced weight loss. Weight was monitored weekly, with any mice losing more than 20% body weight overall or 10% body weight in 1 week required to be sacrificed.

### Transmission electron microscopy

Ultrastructural investigation of the dexamethasone-induced ocular hypertension model and the Tg-MYOC^Y437H^ model was performed by transmission electron microscopy in four pairs of mouse eyes. Eyes were enucleated and immersion-fixed in Karnovsky’s fixative [2.5% paraformaldehyde (PFA), 0.1 M cacodylate, 2.25% glutaraldehyde, and dH_2_O] for 1 hour. Eyes were then removed from fixative, and the cornea was pierced using a 30-gauge needle (BD Microlance 3, Becton Dickinson). Eyes were placed back into fixative overnight at 4°C, washed 3 × 10 min, stored in 0.1 M cacodylate, and sent for electron microscopy analysis. Here, the eyes were cut meridionally through the center of the pupil, the lens was carefully removed, and the two halves of each eye were embedded in Epon. Semi-thin sagittal and then ultrathin sections of SC and TM were cut from one end of each half, and then the other approximately 0.2 to 0.3 mm deeper. The location of the superficial and deeper cut ends was alternated for the second half of the eye such that all four regions examined were at least 0.2 to 0.3 mm distant from one another. The ultrathin sections contained the entire anterior posterior length of the inner wall and the TM. In four regions of each eye, we measured the length of optically empty space immediately underlying the inner wall endothelium of SC. We also measured the inner wall length in contact with ECM, including basement membrane material, elastic fibers, or amorphous material. The optically empty length divided by the total length (optically empty ECM lengths) was calculated and defined as the percentage of optically empty length for that region. All measurements were performed at ×10,000 magnification, with each region including approximately 100 individual lengths of ECM or optically empty space.

### Regional outflow analysis by fluorescent tracers

Fluorescent microspheres (FluoSpheres, carboxylate-modified, 0.1 μm diameter) were injected into the anterior chamber using intracameral injection methods. Ten million microspheres were loaded in 1.5 μl and injected. The animals were left to recover, and 24 hours later, the eyes were enucleated and the anterior chamber was dissected. The cornea was flat-mounted onto poly-lysine slides with the epithelium face up. Slides were mounted, let dry, and imaged en face. Image processing methods were used to segment each eye into 50 sectors and, for each sector, to determine if FluoSpheres were present and to calculate the mean fluorescent intensity. High- and low-flow regions were arbitrarily defined on the basis of whether they were above or below the mean sector intensity.

### Tonometric IOP measurement

For analysis of the weekly change in murine IOP, measurements were performed by rebound tonometry (TonoLab, Icare). Mice were anesthetized with 3% isoflurane at 1 liter/min for 2 min in an induction chamber and then moved to a head holder delivering isoflurane at the same rate. At 3 min after induction of anesthesia, three IOP measurements (constituting an average of six readings each) were made at three different time points in both eyes (eye 1: minutes 3, 5, and 7 after anesthesia; eye 2: minutes 4, 6, and 8 after anesthesia). Data were tested for normality and in these datasets followed a nonparametric distribution. Thus, the median IOP measurement at each time point was used to fit a line and interpolate IOP at minute 5 to allow for measurement of IOP in both eyes while accounting for the IOP-lowering effect of anesthesia over time (*35*). Wilcoxon signed-rank tests were used to determine statistical significance between paired groups such as between contralateral eyes or across time points of the same eyes. Wilcoxon rank sum tests were used to compare independent groups of eyes from one cohort to another, such as when comparing a group of control eyes in hypertensive animals to a group of control eyes in normotensive animals. For the dexamethasone-induced glaucoma mouse model and controls, additional statistical tests on IOP measurements were performed to validate (a) the model [AAV-iGFP treatments were significantly different between hypertensive and normotensive mice at the final time point (*P = 0.015), and AAV-iGFP treatment was significantly different from AAV induction to the final time point (*P = 0.011) given the acute nature of this model] and (b) the effect of the AAV-iMMP3 treatment [AAV-iMMP3–treated hypertensive eyes were not significantly different from normotensive control eyes (*P* = 0.7), AAV-iMMP3–treated hypertensive eyes did not significantly increase in IOP after viral induction unlike contralateral controls (*P* = 0.77), and the IOP change between baseline and final time point was significantly lower in treated eyes (*P = 0.01)].

For the Tg-MYOCY437H mouse model, additional statistical tests on IOP measurements were performed to validate (a) the model [AAV-iGFP–injected control eyes were significantly different between hypertensive and normotensive mice at the final time point (*P = 0.017)] and (b) the effect of the AAV-iMMP3 treatment (AAV-iMMP3–treated hypertensive eyes were not significantly different from normotensive control eyes (*P* = 1), AAV-iMMP3–treated hypertensive eyes significantly decreased in IOP after viral induction unlike contralateral controls (*P* = 0.012), and the IOP change between baseline and final time point was significantly lower in treated eyes (*P = 0.0006)].

### MMP-3 quantification

MMP-3 concentration from media, cell lysate, or aqueous samples was assayed by ELISA or Western blot. The total human MMP-3 Quantikine ELISA kit (R&D Systems, DMP300) was used according to the manufacturer’s protocol. Cell culture samples were harvested 48 hours after transfection and diluted 1:3 for plasmid-treated supernatants and 1:200 for AAV-treated supernatants. Aqueous samples were applied to the ELISA plate neat. Western blot was also used for AAV-treated samples immunoblotted against anti-human MMP-3. Protein samples were loaded onto a 10% SDS-PAGE gel at 30 to 50 μg per well. Proteins were separated by electrophoresis over the course of 90 min at constant voltage (120 V) under reducing conditions and subsequently electrotransferred onto methanol-activated polyvinylidene difluoride membranes at constant voltage (12 V). Membranes were blocked for 1 hour at room temperature in 5% nonfat dry milk and incubated overnight at 4°C with a rabbit primary antibody to MMP-3. Blots were washed 3× 5 min in TBS and incubated at room temperature for 2 hours with horse radish peroxidase–conjugated anti-rabbit secondary antibody (Abcam). Blots were developed on a blot scanner (C-Digit, LI-COR). Ponceau staining was used as a loading control for supernatant samples, and densitometry was performed on resulting bands using ImageJ.

Codon-optimized MMP-3 mRNA levels were determined by qPCR using the following primers: forward = “AGCCATCAGCGACAAGGAAA,” reverse = “GCGTCGATCTTGGAGTCGAT.” RNA was isolated from HCECs using an RNA isolation kit (Omega Biotek). A single SYBR mastermix (Qiagen) and consistent thresholding was used across plates. Samples were normalized to their glyceraldehyde-3-phosphate dehydrogenase (GAPDH) signal, and ΔΔCTs were calculated.

Enzymatic activity of MMP-3 was quantified using fluorescence resonance energy transfer using an activity assay kit (Abcam, ab118972) as per the manufacturer’s instructions. Results are presented as milliunits per milliliter, where 1 unit is defined as the amount of enzyme that will generate 1 μmol of unquenched substrate per minute at room temperature.

### Immunohistochemistry

Globes were marked at the 12:00 position with a suture in the limbal conjunctiva and enucleated. Globes were injected intravitreally with approximately 200 μl of fresh 4% PFA to reinflate the globe and then submerged in 4% PFA. After 6 hours, eyes were transferred to PBS with 0.05% sodium azide in a filled container, capped, and stored a 4°C. Anterior segments were placed in optimum cutting temperature compound and frozen in isopropanol bathed in liquid nitrogen. Embedded tissues were sectioned sagittally in 50-μm sections. Sections were air-dried for 1 hour before incubation with 5% normal goat serum for 1 hour at room temperature. Sections were subsequently incubated with a 1:1000 dilution of chicken anti-GFP antibody (Abcam, ab13970) overnight (16 hours) at 4°C. Sections were washed three times with PBS and incubated with a goat anti-chicken–Alexa Fluor 488 (Abcam, ab150169) for 2 hours at room temperature. Sections were washed twice with PBS, counterstained with 4′,6-diamidino-2-phenylindole (DAPI) (1:5000 dilution) for 45 s, washed once more, and mounted with a coverslip using hydromount (National Diagnostics). Images were obtained using a Zeiss confocal microscope.

### Ocular health assays

Corneal pachymetry was performed at designated time points using an Accutome AccuPach 5 ultrasound pachymeter. A mean pachymetry measure in micrometers was obtained from a series of four successive measures in each eye. Specular microscopy was performed at the same time points with a Tomey EM-3000 specular microscope to evaluate the integrity of the corneal endothelium. The number of analyzed endothelial cells, density, and a range of cell dimensions were quantified. Immediately after aseptic preparation of the eyes, aqueous humor (~100 μl) was collected with a 0.3-ml insulin syringe with a 31-gauge needle and aliquoted to two samples (2 × 50 μl). Aqueous humor aliquots were then transferred to prelabeled cryotubes and stored at −70°C until ELISA was undertaken.

A total of 21 tolerability measures were performed every 2 weeks throughout the study and scored according to severity using the Hackett-McDonald scoring method. The scoring criteria used in this study have been appended to the Supplementary Materials. Each parameter was scored from 0 to 2, 3, or 4 as noted in the scoring method. Last, each animal was scored out of a total possible score of 73 across all measures.

### Ex vivo measurement of outflow facility in whole murine eyes

Outflow facility measurements with iPerfusion were carried out as described previously (*36*). Mice were culled by cervical dislocation, and eyes were enucleated immediately and stored in PBS at room temperature to await perfusion (~20 min). Both eyes were perfused simultaneously using two independent perfusion systems. Briefly, each eye was affixed to a support using a small amount of cyanoacrylate glue and submerged in a PBS bath regulated at 35°C. The eye was cannulated via the anterior chamber with a 33-gauge beveled needle (NanoFil, #NF33BV-2, World Precision Instruments) under a stereomicroscope using a micromanipulator. The iPerfusion system comprises an automated pressure reservoir, a thermal flow sensor (Sensirion, SLG150), and a wet-wet pressure transducer (Omegadyne, PX409) to apply a desired pressure, measure flow rate out of the system, and measure the IOP, respectively. The perfusate was PBS containing Ca^2+^, Mg^2+^, and 5.5 mM glucose, which was filtered through a 0.22-μm filter (VWR International) before use. Following cannulation, eyes were perfused for 30 min at 8 mmHg to allow the eye to acclimatize. Subsequently, nine discrete pressure steps were applied from 4.5 to 21 mmHg, while flow and pressure were recorded. For each step, stability was defined programmatically, and 5 min of data was acquired for each step, filtered using a Savitsky-Golay filter of 60 s and averaged. A power law model was fit to flow-pressure data to account for the pressure dependence of outflow facility in ex vivo mouse eyes. The standardized analytical methodology was applied to determine the treatment effect, while accounting for measurement uncertainties, and statistical significance was evaluated using a paired weighted *t* test (*32*, *36*).

### In vivo measurement of outflow facility in nonhuman primates

At 0 hour on the day of dosing, outflow dynamics were quantified with the iPerfusion system. The system delivered sterile PBS with 5.5 mM glucose with or without MMP-3 (5 ng/ml) to the anterior chamber at the desired pressure and recorded the flow rate of fluid entering the eye. Before perfusion, animals were fasted for ~12 to 14 hours, then sedated with ketamine (10 mg/kg, intramuscularly), and brought to the surgical preparation room. They received atropine sulfate (0.02 to 0.04 mg/kg, intramuscularly), preoperatively, and subsequently every 4 hours throughout anesthesia. Each monkey had a peripheral catheter placed for administration of intravenous fluids (fluid rate, 3 ml/kg per hour). The animal was moved to the surgical suite where it was anesthetised with isoflurane and placed on a ventilator to stabilise breathing rate and oxygen consumption. A heating pad and warm air flow (Bair Hugger) were used to maintain constant body temperature. An electrocardiogram was connected to monitor vitals. Topical local anesthesia was administered (0.5% proparacaine), and eyes were disinfected with 5% Betadine and rinsed with sterile saline. Intracameral cannulations were placed (using a 25-gauge 0.5-inch needles for each eye) 2 mm anterior to the limbus in the temporal quadrant, targeting the central aqueous cavity before infusion. This was achieved with the aid of specula and needle adaptors mounted to flexbars. One drop of GenTeal and eye lubricant ointment was placed in each eye, and the perfusion system was then used to measure flow at a range of physiological levels over a period of approximately 90 min. Both eyes were perfused simultaneously on duplicates of the system. Once the spontaneous IOP (*S*_0_) was determined, the applied pressure was increased by 5 mmHg for an acclimatization period of 1 hour. Pressure was then returned to *S*_0_, and a facility measurement was taken, consisting of 60 s of stable flow readings at 12 different pressure steps of approximately 10 to 15 min per step. Steps that exhibited a significant drift in pressure and flow were omitted, and the two-step analysis method of Bárány was performed to calculate facility for each combination of two sequential steps (*37*). The average of all facility values for each eye and corresponding 95% confidence intervals were calculated. At the end of perfusion, the cannulae were removed and 50 μl of aqueous humor was collected from each eye followed by topical administration of Vigamox (0.5% moxifloxacin hydrochloride) and meloxicam (0.1 mg/kg, subcutaneously) was administered postoperatively for analgesia. After completion of the procedure, the monkey was monitored until full recovery from anesthesia was observed.

### Ex vivo measurement of outflow facility in human donor anterior segments

Eyes were received approximately 24 hours postmortem. Eyes were removed from packaging and sprayed with povidone iodine solution as a disinfectant. Eyes were placed on a surgical drape in a laminar flow hood atop a foam pad, which had a hemisphere cut from the surface. This aided in keeping the eye stable for the initial incisions. Tools were sterilized beforehand using 1% Virkon and 70% ethanol washes. The fluid lines were connected to the eye bath through the incubator, and all lines were filled with media and closed off on the external side so that no bubbles could be introduced once the eye was secured. An initial cut was made using a curved surgical blade, bisecting the eye approximately in half about its circumference. Once initial cuts were made, a dissecting scissors was used to cut around the circumference, separating the anterior chamber from the posterior cup. The lens was carefully removed so as not to disturb the iris. This anterior segment was placed over the eyebath chuck while submersed in human endothelial serum–free media supplemented with FGF-β2 and penicillin-streptomycin. Any bubbles inside the eye were carefully squeezed out, and the segment was oriented on the chuck. Long screws and screw posts were used to secure the clamp over the eye, creating a tight seal between the eye and chuck to prevent any leaks. Once mounted, the incubator was sealed and transferred to the iPerfusion case and fluidics, electronics, and gas systems were connected. Eyes were allowed to stabilize at 12 mmHg, and a facility measurement was then taken as a baseline. This was performed over multiple pressure steps with multiple readings at 12 mmHg to account for the time-dependent effect on facility. Once a measurement was taken, an exchange could then be performed to exchange the contents of the anterior chamber with MMP-3 (5 ng/ml). Syringe pumps were used for this procedure, with exchange performed at 100 μl/min for a total of 6 ml, to include not only the volume of the anterior chamber but also the fluid lines connecting to it as well as error. A second facility measurement was made, and the paired difference between baseline and post-exchange was calculated for each eye.

## Notes

View/request a protocol for this paper from *Bio-protocol*.
