# Supplementary material for: Matrix metalloproteinase-3 (MMP-3)–mediated gene therapy for glaucoma
Source: Sci Adv. 2023 Apr 19;9(16):eadf6537. doi: 10.1126/sciadv.adf6537 (PMC10115410; doi:10.1126/sciadv.adf6537)
Supplement: Supplementary file 1 — Figs. S1 to S5 Table S1 [file sciadv.adf6537_sm.pdf]

Science Advances
AAAS

Supplementary Materials for

Matrix metalloproteinase-3 (MMP-3)–mediated gene therapy for glaucoma

Jeffrey O'Callaghan *et al.*

Corresponding author: Matthew Campbell, matthew.campbell@tcd.ie

Sci. Adv. **9**, eadf6537 (2023)
DOI: 10.1126/sciadv.adf6537

This PDF file includes:

Figs. S1 to S5
Table S1

Table S1. Regulatory Element Optimisations

**Plasmid ID**	**Promoter**	**ORF**	**PolyA**	**Secondary Elements**	**Plasmid Length (bp)**	**Genome Length (bp)**
Native	CMV	Native MMP3	HGH-	CMVa-bglobinB intron	5325	3169
Opt3	CMV	MMP3 (Opt3)	HGH	CMVa-bglobinB intron	5325	3169
376137	CAG	MMP3 (Opt3)	BGH	CBA intron, WPRE	6412	3777
376138	CAG	MMP3 (Opt3)	RbGlobin	WPRE	6412	4256
376640	CAG	MMP3 (Opt3)	RbGlobin	CBA intron, 3'UTR α-globin	6412	4256
376644	CMV	MMP3 (Opt3)	HGH	CMVc intron Expression enhancement element	6412	4256
376645	CAG	MMP3 (Opt3)	HGH	Expression enhancement element	6412	4256
376646	CMV	MMP3 (Opt3)	RbGlobin	WPRE, Expression enhancement element	6412	4256
376647	CMV	MMP3 (Opt3)	HGH	CMVa-β globinB intron, WPRE	5914	3758

Figure S1

A

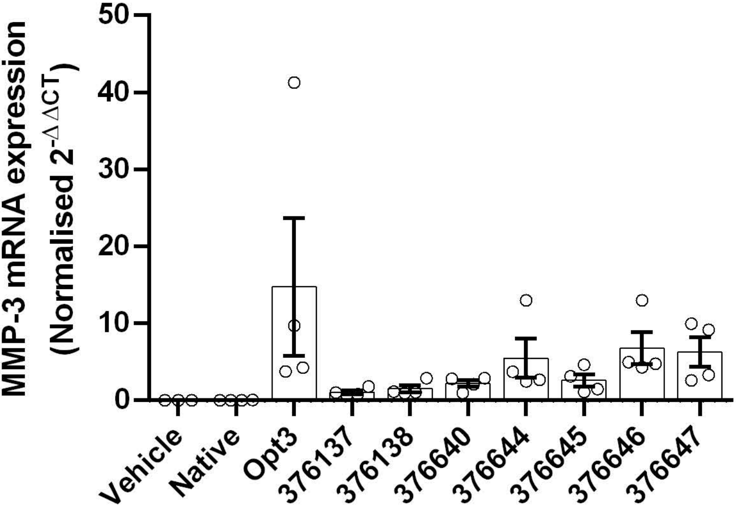

MMP-3 mRNA expression (Normalised $2^{-\Delta\Delta CT}$)
50
40
30
20
10
0
Vehicle
Native
Opt3
376137
376138
376640
376644
376645
376646
376647

B

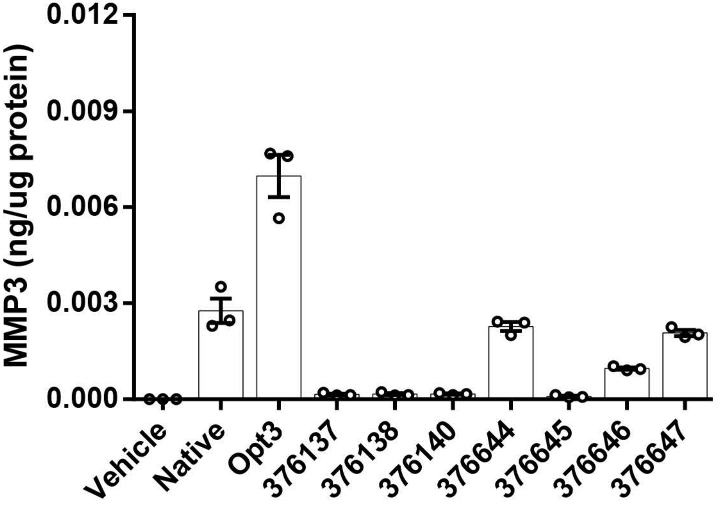

MMP3 (ng/ug protein)
0.012
0.009
0.006
0.003
0.000
Vehicle
Native
Opt3
376137
376138
376140
376644
376645
376646
376647

Figure S2

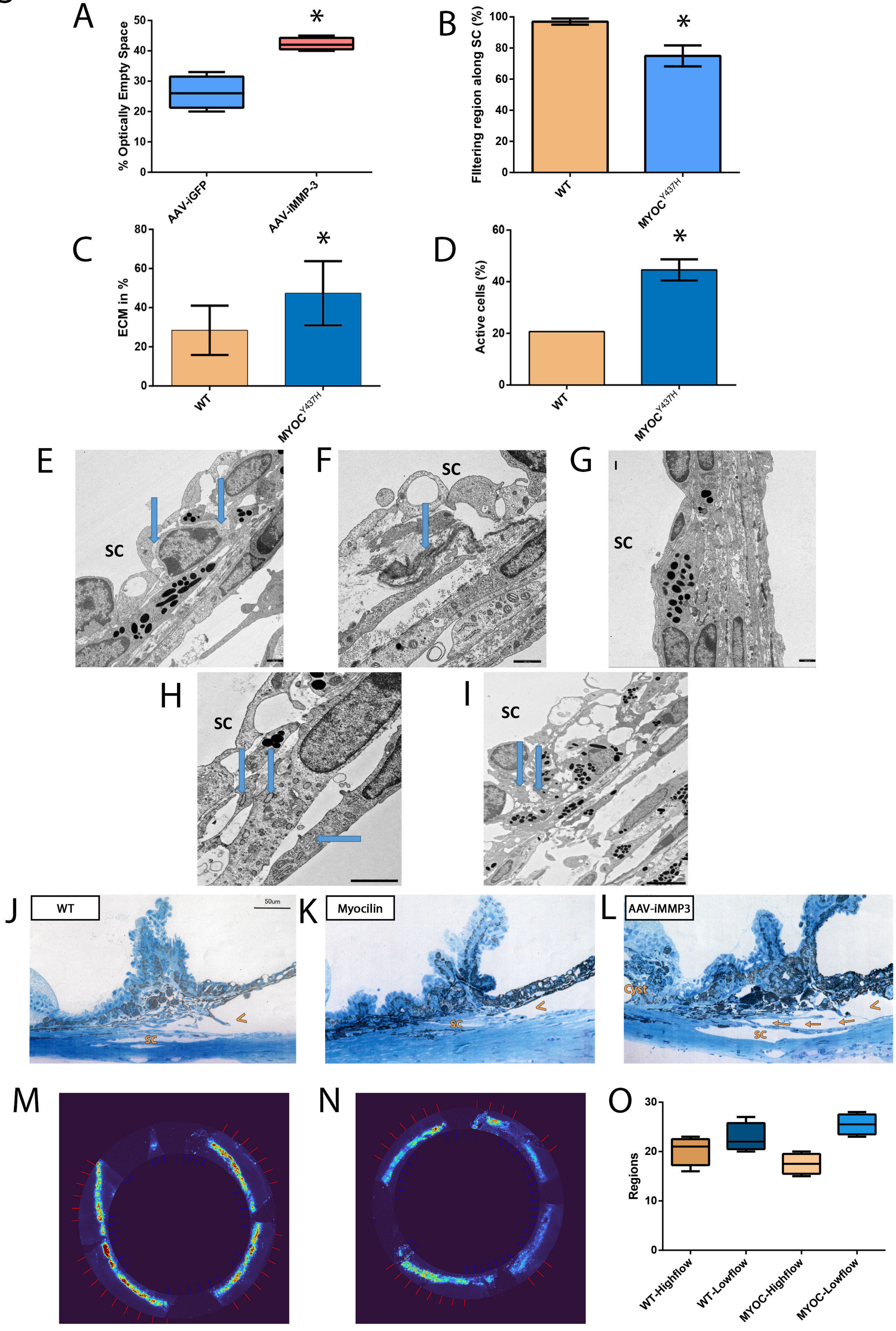

A
% Optically Empty Space
AAV-iGFP
AAV-iMMP-3
B
Filtering region along SC (%)
WT
MYOC Y437H
C
ECM in %
WT
MYOC Y437H
D
Active cells (%)
WT
MYOC Y437H
E
SC
F
SC
G
SC
H
SC
I
SC
J
WT
50um
SC
K
Myocilin
SC
L
AAV-iMMP3
Cyst
SC
M
N
O
Regions
WT-Highflow
WT-Lowflow
MYOC-Highflow
MYOC-Lowflow

Figure S3

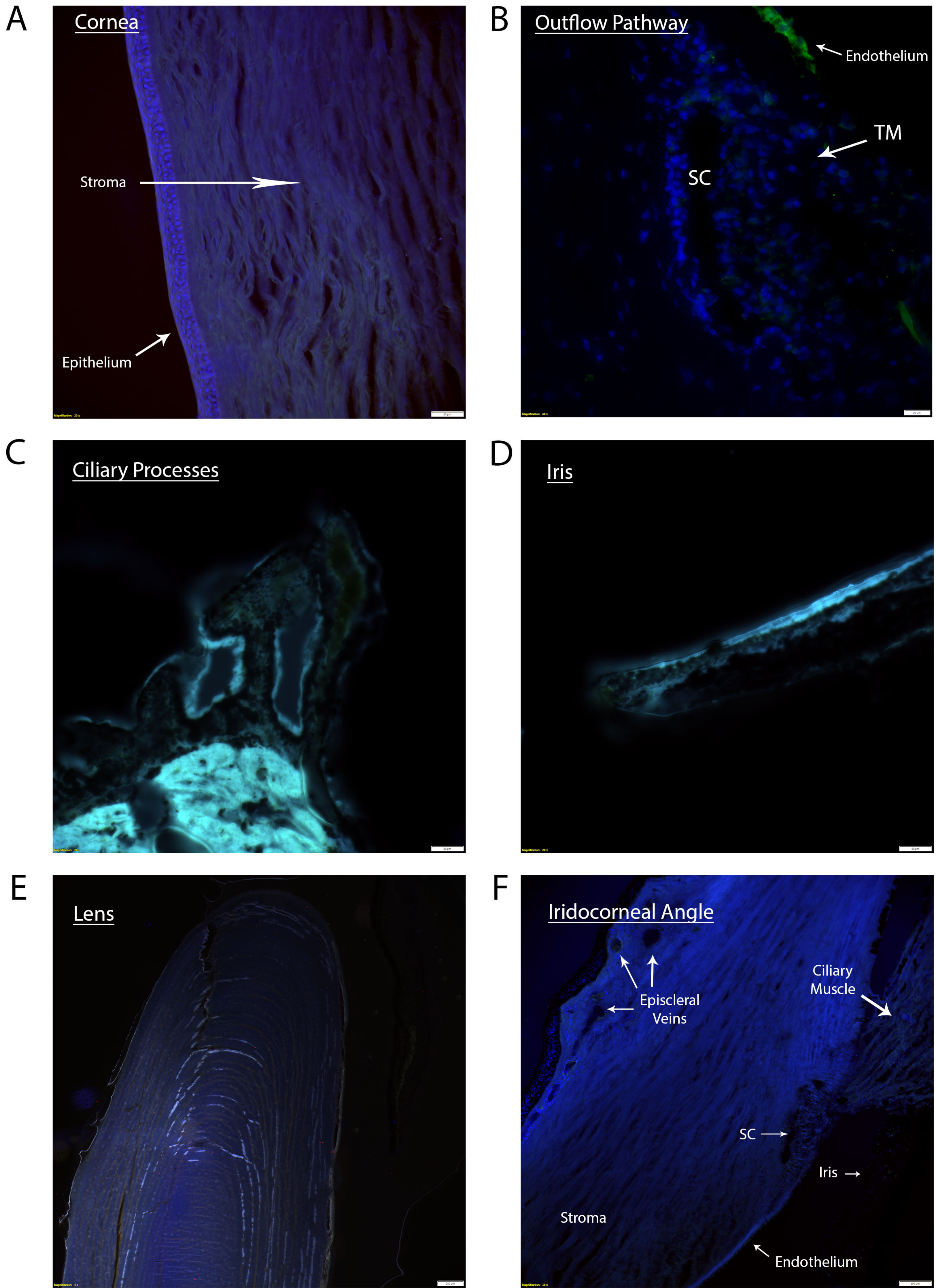
A
Cornea
Stroma
Epithelium
B
Outflow Pathway
Endothelium
TM
SC
C
Ciliary Processes
D
Iris
E
Lens
F
Iridocorneal Angle
Episcleral Veins
Ciliary Muscle
SC
Iris
Stroma
Endothelium

Figure S4

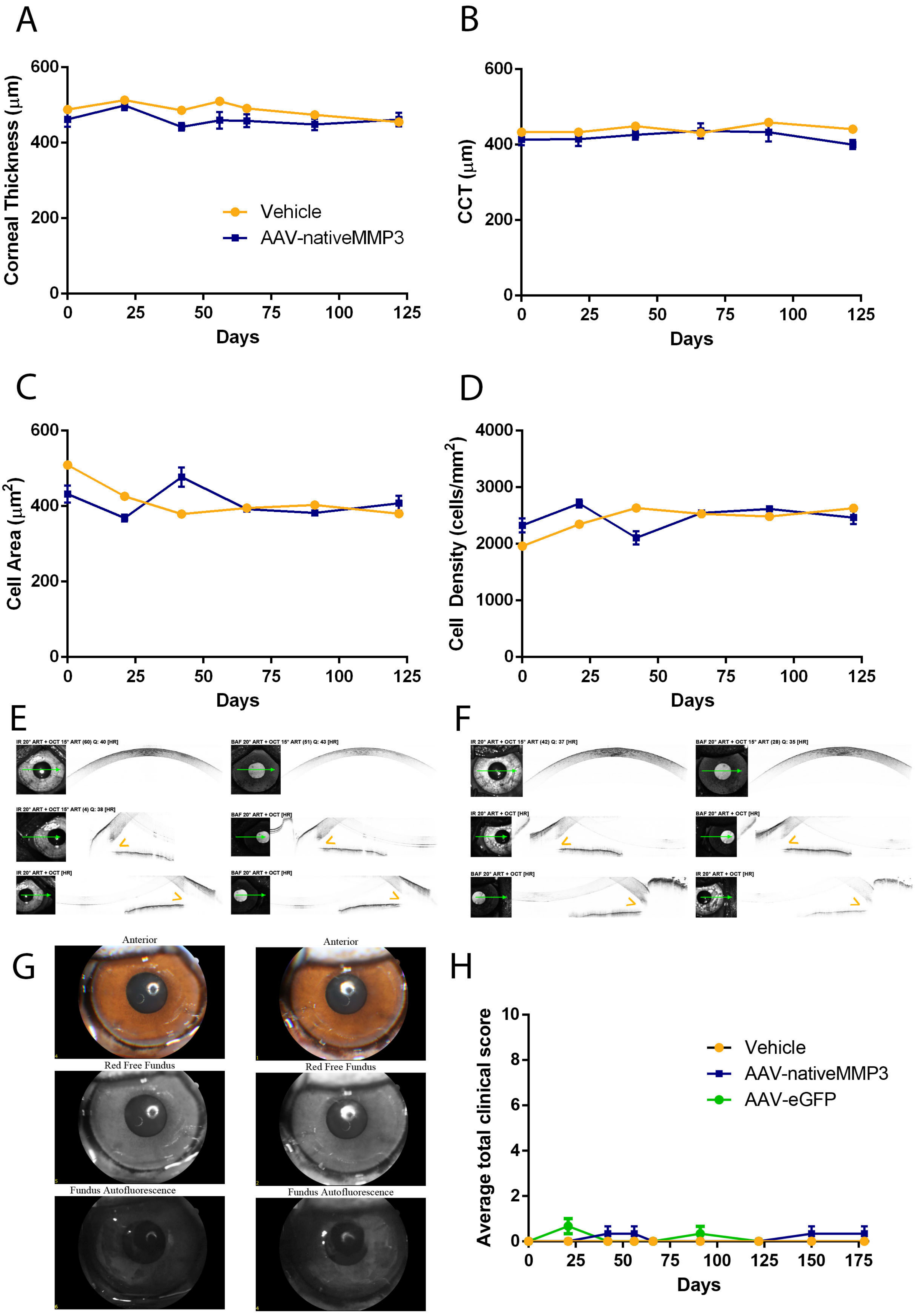

A
Corneal Thickness (μm)
Days
Vehicle
AAV-nativeMMP3
B
CCT (μm)
Days
C
Cell Area (μm²)
Days
D
Cell Density (cells/mm²)
Days
E
F
G
Anterior
Red Free Fundus
Fundus Autofluorescence
H
Average total clinical score
Days
Vehicle
AAV-nativeMMP3
AAV-eGFP

Figure S5

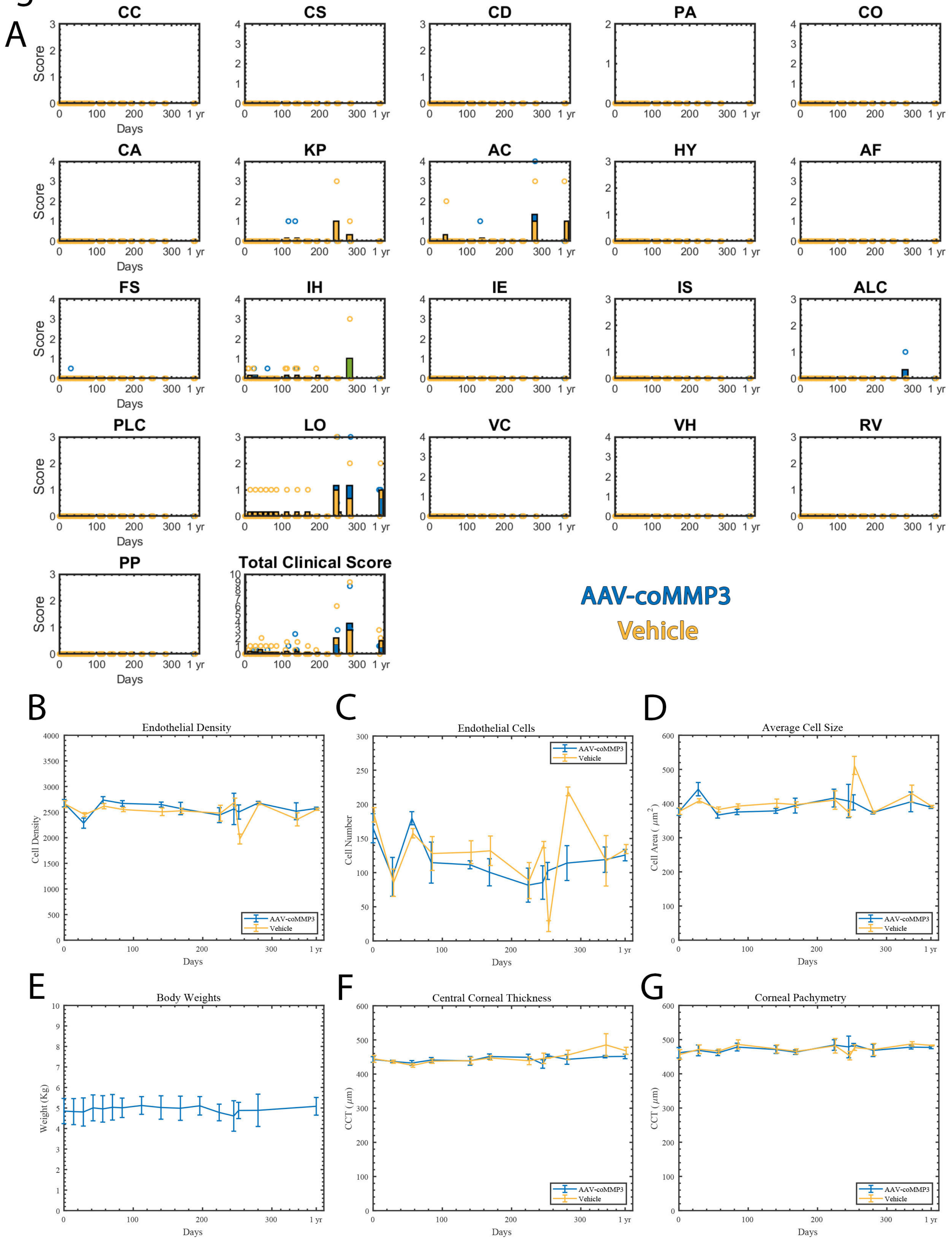

A
CC
CS
CD
PA
CO
CA
KP
AC
HY
AF
FS
IH
IE
IS
ALC
PLC
LO
VC
VH
RV
PP
Total Clinical Score
Score
Days
0
100
200
300
1 yr
AAV-coMMP3
Vehicle
B
Endothelial Density
Cell Density
C
Endothelial Cells
Cell Number
D
Average Cell Size
Cell Area (μm^2)
E
Body Weights
Weight (Kg)
F
Central Corneal Thickness
CCT (μm)
G
Corneal Pachymetry
CCT (μm)

Supplementary Materials

Table S1 depicts the promoter/polyA/other regulatory element optimisation combinations in the various constructs tested for regulatory element optimisation. All plasmids contain the codon optimised version of MMP3, except for the native plasmid, containing unoptimized MMP3.

Figure S1: Regulatory element optimisation
Different plasmid regulatory elements were tested using the Opt3 plasmid, however no improvements on (**A**) RNA or (**B**) protein expression was observed.

Figure S2: Supplementary murine outflow and electron microscopy data
(**A**) Quantified optically empty spaces by TEM in subendothelial region in dexamethasone mice. (**B-D**) quantification of TEM images in myocilin mice. Myocilin mice appear to exhibit reduced filtering regions (**B**), increased ECM along the subendothelial circumference (**C**), and increased active cells (**D**), elevated numbers of Golgi and rER per cell). (**E-I**) TEM feature observations in myocilin mice. Scale bars represent 100 μm. (**E**) Increased ECM deposition (arrows) in the subendothelial region between subendothelial cells. (**F**) Thickening of the elastic fibre sheath (arrow). (**G**) Dense band-like TM with collagen deposits throughout and nearly no optically empty spaces. (**H**) Activated cells had increased numbers of enlarged eER and Golgi (arrows). (**I**) In some places, cell proliferation was apparent, with an increase in subendothelial cells (arrows). (**J-L**) Semithin sections of (**J**) WT, (**K**) myocilin and (**L**) MMP-3 treated myocilin animals. SC length varied more in myocilin animals. SCs were larger with looser TMs in treated eyes, but in some eyes, enlarged uveoscleral pathways (arrows) and peripheral retinal cysts were also present. Representative scale bar for semi thin images in J is 50 μm. (**M**) Fluorescent tracers dispersed in a WT animal and in a (**N**) myocilin control animal. (**O**) The number of high and low flow regions for both animals were quantified, with myocilin animals having a lower number of high flow regions (bounded by red lines), and a greater number of low flow regions on average.

Figure S3: Anterior chamber immunohistochemistry of AAV9-eGFP-injected NHP eyes.
Eyes intracamerally injected with AAV9 expressing GFP were screened for signal throughout the anterior chamber to assess AAV9 tropism. (**A**) Corneal endothelium and stroma demonstrated no signal other than autofluorescence in the stroma. (**B**) Schlemm's canal and trabecular meshwork exhibited no GFP signal, while the corneal endothelium presented a signal, as described in Figure 4. (**C-E**) The ciliary processes, iris and lens showed only autofluorescent signal, demonstrated by a turquoise colour to indicate overlap of DAPI and GFP channels. (**F**) A low magnification image of the iridocorneal angle, displaying no evidence of GFP signals in stroma, iris, ciliary muscle fibres or the Schlemm's canal. A weak signal is apparent in the endothelium, which is visualised more clearly on higher magnification. In this animal alone, marginal GFP signal can be observed in some of the episcleral veins. SC = Schlemm's canal. TM = trabecular meshwork. Scale bars are presented on each image independently.

Figure S4: Preliminary AAV9 safety data
Non-human primate eyes intracamerally injected with AAV9 expressing either MMP-3 or eGFP were screened for several clinical safety measures. Corneal health was a primary concern, and so central corneal thickness was measured by both pachymetry (**A**) and specular microscopy (**B**). Corneal endothelial cell area (**C**) and cell density (**D**) were also monitored. Representative anterior chamber OCTs at day 122 are presented for the OD (**E**) and OS (**F**)

eye. There is no evidence of corneal swelling/shrinkage and iridocorneal angles remain open (orange angles). (**G**) Anterior chamber imaging indicates no abnormalities in either treated or control eye. (**H**) Total clinical score was averaged across animals for each treatment.

Figure S5: Detailed safety data for AAV9-coMMP3

(**A**) 6 non-human primates were intracamerally injected with AAV-coMMP3 in one eye, with a vehicle injection in the contralateral eye. Over the course of 1 year, clinical safety scores were monitored, including conjunctival congestion, conjunctival swelling, conjunctival discharge, pannus, corneal opacity, corneal opacity area, inflammatory keratic precipitates, aqueous cell, hypopyon, aqueous flare, fibrin strands, iris hyperemia, iris exfoliation, iris synechia, anterior and posterior lens capsule deposits, lens opacity, vitreous cell, vitreous haziness, retinal vasculitis and total score. Scores indicate severity, with the total score out of a possible score of 73. Dots indicate an individual's score and bars indicate mean score at that timepoint. Green bars indicate the same average score for both eyes. Scores were generally low, indicating good tolerance to the AAV. Most positive scores were resolved by the next timepoint, and there were very few occurrences of scores exclusive to treated eyes. (**B-D**) Measures of corneal endothelial cells integrity were also monitored including cell density, cell number and cell size. No changes between eyes or over the course of the study were observed. (**E**) Body weights remained unchanged. (**F-G**) Central corneal thickness was measured by both specular microscopy and pachymetry. No significant differences were observed. In all measures, an outlier is observed in control eyes at day 252, where data appears to change but is measured as 'normal' at the next timepoint.
